# Survival predictors in paraquat intoxification and role of immunosuppression

**DOI:** 10.1016/j.toxrep.2014.06.010

**Published:** 2014-07-18

**Authors:** Keng-Hee Koh, Clare Hui-Hong Tan, Lawrence Wei-Soon Hii, Jun Lee, Laura Lui-Sian Ngu, Alvin Jung-Mau Chai, Chek-Loong Loh, Swee-Win Lam, Lily Mushahar, Tem-Lom Fam, Wan Shaariah Md Yusuf

**Affiliations:** a Nephrology Unit and Department of Medicine, Sarawak General Hospital, Kuching, Malaysia; b Nephrology Unit and Department of Medicine, Miri Hospital, Malaysia; c Medical Department, Sibu Hospital, Malaysia; d Medical Department, Hospital Raja Permaisuri Bainun, Ipoh, Malaysia; e Medical Department, Hospital Sultan Abdul Halim, Sungai Petani, Malaysia; f Nephrology Department, Hospital Tuanku Ja’afar, Seremban, Malaysia

**Keywords:** Paraquat poisoning, eGFR, Inflammation, Immunosuppression, Survival, Acute renal failure

## Abstract

Paraquat poisoning resulted in multiorgan failure and is associated with high mortality. We audited 83 historical cases of paraquat poisoning in past 2 years treated with conventional decontamination and supportive treatment, followed by enrolling 85 patients over a 2 year period into additional immunosuppression with intravenous (i.v.) methylprednisolone and i.v. cyclophosphamide.

Our results showed that age, poor renal function and leucocytosis are the main predictors of fatal outcome. Immunosuppression regime rendered higher survival (6 out of 17 patients (35.3%)) versus historical control (1 out of 18 patients (5.6%)) (*p* = 0.041) in the cohort with admission eGFR < 50 ml/min/1.73 m^2^ and WBC count > 11,000/μL.

In contrast, there was no difference in survival with immunosuppression regime (38 out of 64 patients (59.4%)) compared to historical control (30 out of 52 patients (57.7%)) (*p* = 0.885) in those with eGFR > 50 ml/min/1.73 m^2^ or WBC < 11,000/μL at presentation.

Multivariable logistic regression showed survival probability = exp(logit)/(1 + exp(logit)), in which logit = 13.962 − (0.233 × ln(age (year))) − (1.344 × ln(creatinine (μmol/L))) − (1.602 × ln(rise in creatinine (μmol/day))) – (0.614 × ln(WBC (,000/μL))) + (2.021 × immunosuppression) and immunosuppression = 1 if given and 0 if not. Immunosuppression therapy yielded odds ratio of 0.132 (95% confidential interval: 0.029–0.603, *p* = 0.009).

In conclusion, immunosuppression therapy with intravenous methylprednisolone and cyclophosphamide may counteract immune mediated inflammation after paraquat poisoning and improve survival of patients with admission eGFR < 50 ml/min/1.73 m^2^ and WBC count > 11,000/μL.

## 1 Introduction

Paraquat poisoning could result in multiorgan failure. Besides intestinal decontamination [1], [2], the administration of glucocorticoids and cyclophosphamide has been advocated following the study by Lin et al. [3], [4]. Because of constructive appraisal on the actual efficacy of immunosuppression [5], Lin et al. subsequently performed a randomized controlled trial of 23 patients with paraquat poisoning, with measurement of plasma paraquat levels. The study showed that the mortality rate was 31.3% in the treatment arm versus 85.7% in the control arm (*p* = 0.0272) [6]. Another study done in Iran showed similar trend in outcome [7] favouring the use of cyclophosphamide. Nevertheless, to our knowledge, there is not yet any study identifying the specific group that may benefit most from immunosuppression therapy. This in fact is an important piece of information, because one has to ascertain the potential benefit for each patient based on their clinical profile and decides the more suitable modality of treatment, whether to utilize larger dose of immunosuppression or to omit immunosuppression therapy.

## 2 Methods

This is a multicentre clinical trial performed in Ministry of Health Hospitals in Kuching, Miri, Sibu, Ipoh, Sungai Petani and Seremban cities.

The inclusion criteria were:

1. History of recent paraquat ingestion within 3 days prior to admission.
2. Positive urine paraquat test, or presence of any feature of systemic paraquat toxicity involving kidney, liver or lungs.

We excluded those subjects who were pregnant.

All patients were treated with intestinal decontamination (Appendix I) (23) and IV hydration. We enrolled 85 cases of paraquat poisoning in Years 2011 and 2012 into an immunosuppression protocol (Appendix II), comprising of IV methylprednisolone (1 g/day) for first 3 days (adjustment if needed in liver impairment) and IV cyclophosphamide (15 mg/kg/day) for first 2 days (adjustment if needed in acute kidney failure).

Their clinical profile and outcome were compared with historical cohort of 83 cases of paraquat poisoning in the past 2 years (Years 2009–2010).

This study was approval by Malaysian National Medical Research Ethical Committee (NMRR-11-587-9673) and informed consents were taken from patients. Outcome was verified by clinical notes and follow-up phone calls.

Paraquat was tested qualitatively with sodium bicarbonate and sodium dithionite. We estimated eGFR using the MDRD formula [10].

Our approaches in the analysis were:

a) Compare the clinical profile between subjects with immunosuppression therapy versus historical cohort.
b) Identify the predictors for survival.
c) Evaluate if these survival predictors affect the efficacy of immunosuppression in terms of survival.

The statistical data were analysed using Microsoft excel and SPSS 15 (Statistical Package for Social Science, SPSS Inc., Chicago, IL).

Only patients with complete data were included for analysis to derive the final output for statistical tables and figures.

Kolmogorov–Smirnov test was initially used to determine whether the data is in statistical normal distribution and subsequently logarithm transformation would be performed as necessary [11]. These would be followed by appropriate parametric or non-parametric test as well as parameter description: mean ± standard deviation.

Univariate analysis was performed with parametric test (e.g., Student's *t*-test, ANOVA) for survival comparison in data with statistical normal distribution and geometric transformation was performed as necessary. Factors that significantly affect the predictor and survival were analysed with ANCOVA test.

Chi square test and Fisher's exact test will be utilized according to the standard statistical procedure.

Finally we apply logistic regression to identify the risk predictors and use these factors to identify the patients that benefit best from immunosuppression.

## 3 Results

### 3.1 Comparison of baseline clinical parameters between the subjects with immunosuppression therapy and historical cohort

There were no significant differences in clinical parameters on admission between the two groups (Table 1).

**Table 1:** Univariate analysis: comparison of clinical parameters during admission between subjects in immunosuppression arm and historical cohort.

		*N*	Mean immunosuppression	Historical cohort	*p*-value
Age	Year	159	30 ± 14	34 ± 17	NS
Gender a	F:M	168	33:52	33:50	NS
Vomit a	Y:N	127	67:9	43:8	NS
Duration from paraquat ingestion to admission	h	153	10.2 ± 15.8	17.0 ± 41.7	NS
Amount of paraquat concentrate ingested	ml	143	164 ± 224	127 ± 162	NS
Creatinine	μmol/L	162	153 ± 175	176 ± 152	NS
Initial rise in creatinine	μmol/L per day	121	113 ± 136	78 ± 179	NS
eGFR	ml/min/1.73 m^2^	154	80 ± 43	64 ± 45	NS
Urea	mmol/L	167	5.9 ± 6.5	7.1 ± 9.3	NS
Total bilirubin	μmol/L	151	15.3 ± 12.6	28.0 ± 53.8	NS
Conjugated bilirubin	μmol/L	81	7.8 ± 14.2	3.8 ± 5.0	NS
AST	U/L	141	108 ± 366	60 ± 77	NS
ALT	U/L	150	82 ± 244	46 ± 91	NS
WBC	,000/μL	156	12.6 ± 4.3	14.7 ± 7.4	NS b
HCO_3_	mmol/L	127	20.9 ± 4.8	19.8 ± 5.1	NS
PaO_2_	mmHg	126	94 ± 31	88 ± 28	NS

*Abbreviation*: NS, not significant.

Univariate analysis was performed with Student's *t*-test.

a Chi square test was performed for gender in univariate analysis.

b Logarithm transformation was performed to achieve Gaussian normal distribution, because of two subjects with extreme leucocytosis > = 35,000/μL.

### 3.2 Identification of survival predictors

**Fig. 1:**
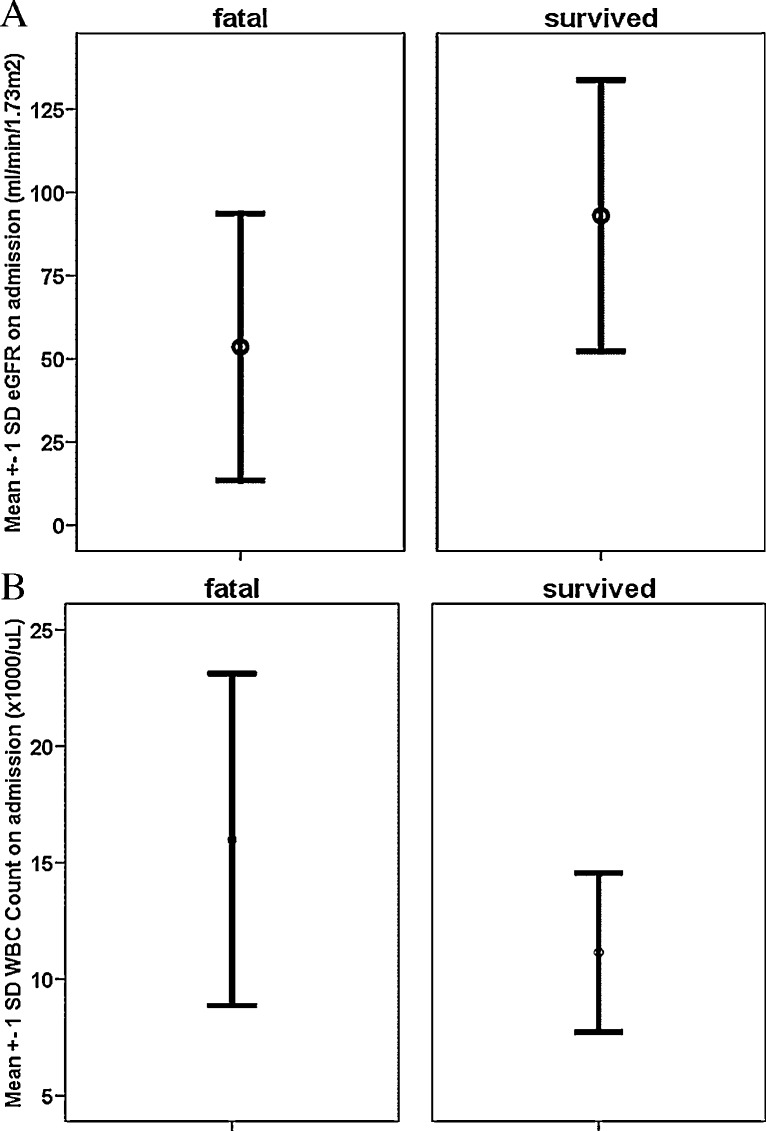
(A) Estimated glomerular filtration rate (eGFR) on admission. Standard deviation (SD) of 81 fatal cases versus 73 survived cases was shown. (B) White blood cell (WBC) count on admission. Standard deviation (SD) of 78 fatal cases versus 78 survived cases was shown.

Table 2 and Fig. 1A and B showed overall better survival in patients with higher eGFR (estimated glomerular filtration rate), low serum creatinine, slower creatinine rise, lower white blood cell (WBC) count, higher serum bicarbonate (HCO_3_), besides traditional predictors of younger age and smaller amount of paraquat ingestion.

**Table 2:** Univariate analysis: comparison of clinical parameters during admission in survived and fatal patients in all subjects.

		*N*	Mean survived	Fatal	*p*-value non-adjusted	*p*-value adjusted a	*p*-value adjusted b
Age	Year	159	29 ± 15	35 ± 15	0.011		
Gender c	F:M	168	41:41	21:65	0.005	0.022	0.294
Vomit c	Y:N	127	50:10	60:7	0.304		
Duration from paraquat ingestion to admission	h	153	9.1 ± 16.4	17.4 ± 39.6	0.093		
Estimated amount of paraquat concentrate ingested	ml	143	89 ± 139	201 ± 229	<0.001	0.002	
Creatinine on admission	μmol/L	162	103 ± 76	221 ± 201	<0.001	<0.001	<0.001
Rise in creatinine within 24 h	μmol/L per day	121	30 ± 71	178 ± 187	<0.001	<0.001	<0.001
eGFR	ml/min/1.73m^2^	154	93 ± 41	54 ± 40	<0.001	<0.001	<0.001
Urea	mmol/L	167	4.6 ± 3.5	8.4 ± 10.3	0.002	0.003	0.034
Total bilirubin	μmol/L	151	15.3 ± 12.6	28.0 ± 53.8	0.048	0.073	
Conjugated bilirubin	μmol/L	81	4.8 ± 9.2	7.2 ± 12.6	0.325		
AST	U/L	141	48 ± 79	122 ± 373	0.108		
ALT	U/L	150	39 ± 60	92 ± 261	0.084		
WBC	,000/μL	15.6	11.1 ± 3.4	16.0 ± 7.1	<0.001	<0.001	<0.001
HCO_3_	mmol/L	127	22.2 ± 4.4	19.0 ± 4.8	<0.001	<0.001	<0.001
PaO_2_	mmHg	126	94 ± 27	89 ± 32	0.377		

Unadjusted univariate analysis was performed with Student's *t*-test. Note: male subjects has consumed higher amount of paraquat concentrate than female (192 ± 221 vs 77 ± 129 ml, *p* < 0.001).

a Univariate adjusted analysis with ANCOVA were performed with age, if the unadjusted analysis by *t*-test demonstrated significant differences.

b Univariate adjusted analysis with ANCOVA were performed with age and estimated amount of paraquat consumption, if the unadjusted analysis by *t*-test demonstrated significant differences.

c Chi square test was performed for gender in univariate analysis.

### 3.3 Evaluation of the efficacy of immunosuppression in groups with various survival predicting parameters

Comparing the two groups overall, there was mild survival benefit with 44 over 85 immunosuppression groups (52%), versus 38 over 83 historical controls survived (46%) (*p* = 0.438).

**Fig. 2:**
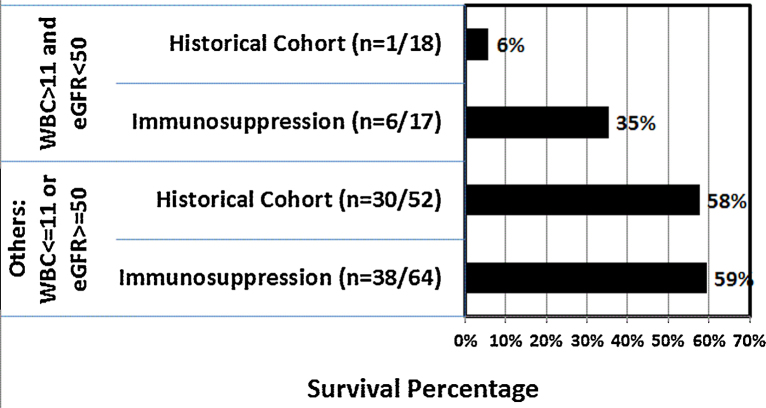
Survival in various cohorts of eGFR and WBC count on admission.

However, in cohort with eGFR < 50 ml/min/1.73 m^2^ and WBC count > 11,000/μL at presentation, immunosuppression regime rendered significantly higher survival rate (6 out of 17 patients (35.3%)) when compared to historical control (1 out of 18 patients (5.6%)) (*p* = 0.041) (Fig. 2).

Nevertheless, there was no difference in survival with immunosuppression regime (38 out of 64 patients (59.4%)) compared to historical control (30 out of 52 patients (57.7%)) (*p* = 0.885) in those with eGFR > 50 ml/min/1.73 m^2^ or WBC < 11,000/μL at presentation.

In Table 3, multivariable logistic regression yielded survival probability = exp(logit)/(1 + exp(logit)),

$$S=\frac{e^{\text{logit}}}{1+e^{\text{logit}}}$$

**Table 3:** Logistic regression models to assess immunosuppression treatment response.

Parameters	Per unit increment	β for survival	β for fatal	Odds ratio	95% CI		*p*-value	*χ*^2^	*p*-value
					Lower bound	Upper bound			
Model with creatinine *R*^2^ = 0.460 (*n* = 87)				All patients					
Intercept		13.962	−13.962				0.001	14.2	0.000
ln(age)	Year	−0.233	0.233	1.262	0.286	5.573	0.759	0.1	0.759
ln(creatinine)	μmol/L	−1.344	1.344	3.835	1.489	9.881	0.005	9.0	0.003
ln(rise in creatinine)	μmol/L/day	−1.602	1.602	4.963	2.290	10.757	0.000	30.4	0.000
ln(white blood cell)	,000/μL	−0.614	0.614	1.847	0.343	9.936	0.475	0.5	0.474
Immunosuppression		2.021	−2.021	0.132	0.029	0.603	0.009	8.5	0.003
Overall model								53.6	<0.001

Model with eGFR *R*^2^ = 0.437 (*n* = 86)				All patients					
Intercept		5.923	−5.923				0.102	2.8	0.093
ln(age)	Year	−0.203	0.203	1.226	0.294	5.110	0.780	0.1	0.780
eGFR	ml/min/1.73 m^2^	0.014	−0.014	0.986	0.973	0.999	0.030	5.3	0.021
ln(rise in creatinine)	μmol/L/day	−1.383	1.383	3.988	2.032	7.829	<0.001	26.4	<0.001
ln(white blood cell)	,000/μL	−0.775	0.775	2.170	0.414	11.373	0.359	0.8	0.359
Immunosuppression		1.887	−1.887	0.152	0.036	0.637	0.010	8.0	0.005
Overall model								49.3	<0.001

*Abbreviation*: CI, confidence interval.

Probablity of survival = e^logit^/(1 + e^logit^), in which logit = 13.962 − (0.233 × ln(age (year))) − (1.344 × ln(creatinine (μmol/L))) − (1.602 × ln(rise in creatinine (μmol/day))) − (0.614 × ln(WBC (,000/μL))) + (2.021 × immunosuppression) and *R*^2^ = 0.460 and immunosuppression = 1 if given and 0 if not.

Alternatively, logit = 5.923 − (0.203 × ln(age)) + (0.014 × eGFR) − (1.383 × ln(rise in creatinine)) − (0.775 × ln(WBC)) + (1.887 × immunosuppression) and *R*^2^ = 0.437.

Immunosuppression therapy yielded odds ratio of 0.132 versus historical cohort management without immunosuppression (95% confidential interval: 0.029–0.603, *p* = 0.009).

Only 21 subjects have undergone haemodialysis. Among them, 15 subjects have haemodialysis within first day after ingestion of paraquat and 6 survived (40%). In contrast, out of 153 subjects who have no haemodialysis within a day after paraquat ingestion, 77 survived (50%, *p* = 0.445).

## 4 Discussion

Paraquat poisoning results in multiorgan failure with pulmonary fibrosis, acute renal failure, liver impairment and is associated with high mortality. Besides intestinal decontamination [12], immunosuppression has been advocated over the past 1–2 decades by many toxicology experts and has demonstrated potential survival benefits [3], [4], [5], [6], [7], [8], [9]. Subsequently a meta-analysis without serum paraquat level as comparators [8], [9] had shown potential survival benefit with concurrent use of glucocorticoid and cyclophosphamide. However, none of these studies identify the subgroup of subjects that may benefit most from immunosuppression therapy.

As consistent with previous studies [5], our study demonstrated that renal function and WBC at presentation were the key factors that influence outcome. These parameters served as important tools and might potentially act as survival predictors besides the serum paraquat level [12], [13]. Besides these, the traditional markers, i.e., age and amount of paraquat consumption are still relevant factors in survival prediction [14].

Renal function at presentation is an important predictor of survival and we wish to postulate that the significance of renal function might be due to (1) worse renal function at presentation or faster deterioration of renal function signifies greater degree of paraquat intoxication or paraquat induced inflammation. (2) Paraquat is excreted renally and impaired renal function results in reduced excretion of paraquat and greater toxicity. Our study looked at the effect of immunosuppression in the group with poor renal function and high WBC and showed that immunosuppression renders survival benefit in this group of patients, whereas there was no survival differences in the group with better preserved renal function and lower WBC. This suggests that immunosuppression may counteract immune mediated inflammation after paraquat poisoning and this is most significant in those with diminished renal function and leucocytosis. The fact that it did not affect the survival in those with preserved renal function and lower WBC suggests that there may be other mechanism of damage besides immune mediated injury in paraquat poisoning.

Overall in our series, the survival is still not optimal despite immunosuppressive therapy, other treatment modality need to be explored to try to improve survival. As acute renal failure of paraquat poisoning is presumably an oxidative stress disorder [15], desferrioxamine may be useful in its treatment. In oxidative stress, lipid peroxidation may be enhanced by iron radicals, and chelating agent desferrioxamine has been shown to reduce toxicity in animal model [16]. Desferrioxamine was used in the management of a single patient with severe paraquat intoxication, in combination with decontamination, haemodialysis, and *N* acetylcysteine with good outcome [17]. Desferrioxamine was also included in the paraquat poisoning protocol in a study report in Korea [15].

Besides haemoperfusion, as paraquat could be removed by dialysis because of its low molecular weight (257 g/mol), extracorporal elimination might be worthwhile in early phase, including haemodialysis and haemofiltration. Nevertheless, various studies show variable and even contrary results for all these modalities [18], [19], [20]. Wide volume of distribution of paraquat in body tissues 1.2–1.6 L/kg [21] and potential of transient renal function reduction might be the reason of limitation of efficacy in these modalities. Besides, it might be hard to organize haemofiltration and haemoperfusion in good timing in district hospital in rural area.

Besides exploring other treatment options such as desferrioxamine, *N* acetyl cysteine, dialysis or haemofiltration, a change in policy regarding usage of paraquat, dilution of paraquat is also a potential tool to further reduce the mortality [22], [23], [24].

In conclusion, paraquat poisoning is still associated with very high mortality rate with our current treatment regimen. Renal function and leucocyte count on admission may be practical clinical parameters to help predict survival and response to immunosuppression therapy. Immunosuppression with intravenous methylprednisolone and cyclophosphamide might potentially improve patient survival especially in those with eGFR < 50 ml/min/1.73 m^2^ and WBC count > 11,000/μL at presentation.

## Disclosure

All the authors declared no competing interest. This paper has not been published previously in whole or part, except in abstract format.

## Research ID with Medical Research Ethical Committee

NMRR-11-587-9673.

## Grant support

None.

## Transparency document

Transparency document

## Appendix I Paraquat poisoning management. Modified from Sarawak Handbook of Medical Emergencies 2nd and 3rd edition [2] with permission

1. Insert nasogastric tube.
2. Stomach washout as soon as possible.
3. Administer 300 ml of Fuller's earth via NG tube as soon as possible, then 20 ml of Fuller's earth every hour until diarrhoea and passage of Fuller's earth.
4. Magnesium sulphate (Mist alba) 30 ml every 4 h for until diarrhoea and passage of Fuller's earth.
5. Give 50 g of activated charcoal stat, then 25 g 4 h for several days.
6. IV fluids approx. 4–5 L/day (NS and D5%) for the first 24 h, then approx. 3 L/day orally or IV subsequently for several days. In established renal failure, IV or oral fluids should be administered according to the fluid status and urine output.
7. Potassium supplement either IV or orally depending on BUSE.
8. Frusemide 40 mg bd IV or oral for several days.
9. Haemodialysis or charcoal haemoperfusion may be useful if started early, especially within 5–7 h of ingestion.

## Appendix II Immunosuppression protocol for the management of paraquat poisoning

1. *Pulse therapy with IV methylprednisolone (1 g/day) for first 3 days* (adjustment: 500 mg/day if patient weight is <40 kg, or if liver impairment developed).
2. However if IV methylprednisolone is not available upon diagnosis in District Hospital, District Medical Officer could give iv hydrocortisone 200 mg stat and send patient to referral centre for 3 days of IV methylprednisolone.
3. *IV cyclophosphamide (15 mg/kg/day) for first 2 days* (adjustment might be needed if renal impairment developed).
4. If patient developed renal failure or PaO_2_ < 10.7 kPa (80 mmHg), to switch to IV or oral dexamethasone 16 or 24 mg daily after pulse methylprednisolone, and stop when renal function improving and PaO_2_ > 80 mmHg.
5. Consider repeat pulse therapy with methylprednisolone (1 g/day for 3 days) if PaO_2_ was <8.0 kPa (60 mmHg) or patient is deteriorating without other explainable reason. Additional IV cyclophosphamide (15 mg/kg/day for 1 day) could be considered if renal function is normal but respiratory function gets worse.
6. Watch out for concurrent infection.
7. If urine paraquat positive and urine output reduced <100 ml/h even with hydration, start haemodialysis as soon as possible for 4 h, Qb 300 ml/min, Qdialysate 500 ml/min and continue daily haemodialysis until urine paraquat is negative.
8. Immunosuppression should be initiated as early as possible *without* waiting for dialysis.
